# Safety and Efficacy of Hepatic Artery Embolization in Heavily Treated Patients with Intrahepatic Cholangiocarcinoma: Analysis of Clinicopathological and Radiographic Parameters Associated with Better Overall Survival

**DOI:** 10.3390/curroncol30100663

**Published:** 2023-10-18

**Authors:** Sara Velayati, Ahmed Elsakka, Ken Zhao, Joseph P. Erinjeri, Brett Marinelli, Mohamed Soliman, Olivier Chevallier, Etay Ziv, Lynn A. Brody, Constantinos T. Sofocleous, Stephen B. Solomon, James J. Harding, Ghassan K. Abou-Alfa, Michael I. D’Angelica, Alice C. Wei, Peter T. Kingham, William R. Jarnagin, Hooman Yarmohammadi

**Affiliations:** 1 Department of Radiology, Memorial Sloan Kettering Cancer Center, New York, NY 10065, USA; svelayati@wphospital.org (S.V.); elsakkaa@mskcc.org (A.E.); zhaok@mskcc.org (K.Z.); erinjerj@mskcc.org (J.P.E.); marinelb@mskcc.org (B.M.); solimam1@mskcc.org (M.S.); olivier.chevallier@chu-dijon.fr (O.C.); zive@mskcc.org (E.Z.); brodyl@mskcc.org (L.A.B.); sofoclec@mskcc.org (C.T.S.); solomons@mskcc.org (S.B.S.); 2 Department of Vascular and Interventional Radiology, Image-Guided Therapy Center, François-Mitterrand University Hospital, 21079 Dijon, France; 3 Department of Medical Oncology, Memorial Sloan Kettering Cancer Center, New York, NY 10065, USA; hardinj1@mskcc.org (J.J.H.); abou-alg@mskcc.org (G.K.A.-A.); 4 Department of Surgical Oncology, Memorial Sloan Kettering Cancer Center, New York, NY 10065, USA; dangelim@mskcc.org (M.I.D.); weia@mskcc.org (A.C.W.); kinghamp@mskcc.org (P.T.K.); jarnagiw@mskcc.org (W.R.J.)

**Keywords:** hepatic artery embolization, intrahepatic cholangiocarcinoma, tumor response, overall survival

## Abstract

The safety and efficacy of hepatic artery embolization (HAE) in treating intrahepatic cholangiocarcinoma (IHC) was evaluated. Initial treatment response, local tumor progression-free survival (L-PFS), and overall survival (OS) were evaluated in 34 IHC patients treated with HAE. A univariate survival analysis and a multivariate Cox proportional hazard analysis to identify independent factors were carried out. Objective response (OR) at 1-month was 79.4%. Median OS and L-PFS from the time of HAE was 13 (CI = 95%, 7.4–18.5) and 4 months (CI = 95%, 2.09–5.9), respectively. Tumor burden < 25% and increased tumor vascularity on preprocedure imaging and surgical resection prior to embolization were associated with longer OS (*p* < 0.05). Multivariate logistic regression analysis demonstrated that tumor burden < 25% and hypervascular tumors were independent risk factors. Mean post-HAE hospital stay was 4 days. Grade 3 complication rate was 8.5%. In heavily treated patients with IHC, after exhausting all chemotherapy and other locoregional options, HAE as a rescue treatment option appeared to be safe with a mean OS of 13 months. Tumor burden < 25%, increased target tumor vascularity on pre-procedure imaging, and OR on 1 month follow-up images were associated with better OS. Further studies with a control group are required to confirm the effectiveness of HAE in IHC.

## 1. Introduction

Intrahepatic cholangiocarcinoma (IHC) is a rare and aggressive form of liver cancer that originates in the bile ducts within the liver. IHC is the second most common primary liver malignancy after hepatocellular carcinoma and accounts for 10–20% of all primary liver cancers [1]. Surgical resection is currently the only curative treatment option for IHC with 5-year overall survival (OS) rates of 22–44% [2,3]. However, only 15% of the patients are surgical candidates at the time of diagnosis. As with many forms of cancer, the treatment landscape for IHC has evolved over the years, with a growing emphasis on therapeutic modalities that offer improved outcomes and enhanced quality of life for patients. Options for non-surgical candidates include locoregional therapies (LRTs), systemic therapy [4]. LRTs are an innovative and promising approach and are commonly performed with a palliative-intent to relieve symptoms or to preserve and improve quality of life [5]. LRTs include percutaneous ablation, hepatic artery embolization (HAE), transarterial chemoembolization (TACE), transarterial radioembolization (TARE), hepatic artery infusion (HAI) and external beam radiation. Prior studies have demonstrated that TARE and TACE are safe and effective in the treatment of patients with IHC. However, to the best of our knowledge, there have been only a few reports on the efficacy and safety of HAE for IHC [6,7]. Additionally, there is limited information on the prognostic factors that predict better outcome after HAE in these patients.

The aim of this study was to evaluate the safety and efficacy of HAE in treating IHC and to identify prognostic factors affecting the outcome.

## 2. Materials and Methods

Institutional Review Board approval was obtained for this retrospective study. From May 2004 to December 2021, all patients with IHC who were treated with HAE were reviewed.

The diagnosis of IHC was confirmed by histopathological assessment. Patients with histopathological diagnosis of mixed cholangiocarcinoma–hepatocellular carcinoma or with concurrent active cancer other than IHC were excluded. All patients received preprocedural multiphasic CT or MRI to delineate the disease extent and stage of the disease burden. In addition, all patients underwent preprocedural laboratory tests to identify baseline liver function. Exclusion criteria for performing HAE included total serum bilirubin greater than 2.0 md/dL, contraindication to angiography, and when more than 75% of the liver was replaced by tumor. Patients with extrahepatic metastatic disease were treated with HAE only if they met the following inclusion criteria: A. patients who had become chemoresistant and had liver-predominate disease, including those experiencing both intrahepatic and extrahepatic involvement; B. patients with extrahepatic disease whose hepatic disease was progressing while undergoing chemotherapy, with a positive response in their extrahepatic disease; C. patients with liver dominate disease who exhibited systemic toxicity from chemotherapy; or D. treatment for palliative reasons.

Electronic medical records were reviewed for age, gender, date of diagnosis, histopathological tumor grade, number, and type of previous therapies (i.e., surgery, type of surgery and hepatic artery infusion pump placement), medical comorbidities, number of HAE procedures, date of first embolization, and type of embolic material used. The Eastern Cooperative Oncology Group (ECOG) performance status was recorded. Post-embolization adverse events were classified using the Common Terminology Criteria for Adverse Events v5.0. (CTCAE) [8]. Imaging records were reviewed for location of primary tumor (intrahepatic versus extrahepatic), tumor pattern (solitary vs. multifocal) and tumor burden (<25%, 25–50%, >50%), size of largest tumor, presence of extrahepatic metastasis at first presentation and location of metastasis. Tumor vascularity was judged visually by 2 interventional radiologists using the preprocedure CT or MRI images without and with contrast enhancement. In solitary lesions, if more than 50% of the tumor enhanced more than the background in the late arterial phase, the tumor was classified as “hypervascular”. Tumors with only a hypervascular rim were classified based on the degree of enhancement of the tumor and not the rim only. In multifocal lesions, classification was based on the degree of enhancement of the majority of the tumors. For example, if more than 50% of the tumors were hypervascular, that patient was classified in the hypervascular tumor group. If no non-contrast images were available on the pre-embolization CT or MRI, tumor vascularity was evaluated based on angiographic findings. Tumors with obvious tumor blush on digital subtraction angiography (DSA) study were classified as hypervascular. All other tumors were classified as non-hypervascular. Data were compiled into a Health Insurance Accountability and Portability Act of 1996 compliant database.

The HAE technique has been previously described [9]. HAE was performed under general anesthesia or conscious sedation. All patients received one dose of prophylactic intravenous antibiotic (cefazolin or ciprofloxacin if allergic to cefazolin) prior to the embolization. Under standard sterile conditions, femoral access was obtained. A base catheter was advanced under continuous fluoroscopic monitoring into the abdominal aorta. DSA of the hepatic arterial vasculature was performed to delineate the arterial supply of the tumor(s). In select cases, a contrast-enhanced cone-beam computed tomography (CBCT) or computed tomography angiography (CTA)was performed to better identify the tumor-feeding vasculature and ensure tumor coverage. Treatment with either Embospheres™ microspheres (40–120 or 100–300 µm; Merit Medical, South Jordan, UT, USA), Embozene™ microspheres (100 µm; Boston Scientific, Burlington, MA, USA), or Bead Block™ microspheres (100–300 µm; Biocompatibles, Furnham, UK) was delivered under continuous angiographic monitoring until complete arterial stasis was achieved. Particle type and size was selected at the discretion of the interventional radiologist as previously described. Following the procedure, the patients were transferred to the recovery unit and admitted for observation of post-embolization syndrome. Patients with disease in both lobes were treated in two separate sessions. Completion of treatment was defined as the time that embolization of both lobes and all hepatic tumors was completed. In patients with disease limited to one lobe, treatment of all tumors was achieved in one setting and the completion treatment was the time of the first HAE; in patients with bilobar disease, this was defined as the time of the second embolization.

Chemorefractory was characterized by the presence of disease progression, either within the liver or extrahepatic progression, as observed on 3-month follow-up radiographic imaging.

Patients were followed up in 1 month with a multiphasic contrast-enhanced CT or MRI. Modified Response Evaluation Criteria in Solid Tumors (mRECIST) criteria were used to evaluate the treatment response [10]. Objective response (OR) rate was defined as the sum of CR and PR rates. Local progression-free survival (L-PFS) was defined as the time between the first embolization date and the detection of local progression or recurrence at the site of treated tumor(s), or date of death or last alive contact without progression. Overall survival (OS) was defined as the time between the date of first embolization and the date of death or censorship at the date of last follow-up.

Factors that we analyzed for outcome were demographic characteristics, pathological grade of the tumor, prior treatment (no prior treatment vs. surgical resection vs. infusion pump vs. systemic chemotherapy), presence of extrahepatic metastasis at the time of diagnosis, indication for embolization (chemorefractory vs. primary treatment), venous invasion, tumor response as per mRECIST and tumor pattern including solitary vs. multifocal, tumor size, and percentage of liver replaced by tumor.

The Kaplan–Meier method was employed to calculate local tumor PFS and OS and Cox regression analysis was used to determine the influence of tumor grade, tumor size category, tumor response category, and the line of HAE therapy on survival outcomes. Changes in laboratory values were examined with a paired T-test, with adjustment for multiple comparisons performed. All statistical analyses were performed with software (SPSS statistics version 25; EXCEL version 1803).

## 3. Results

### 3.1. Demographic Characteristics

From May 2004 to December 2021, a total of 34 patients with IHC underwent HAE. Patient demographics and clinical characteristics are summarized in Table 1. Mean age was 62 ± 14.03 years (range: 31–92; male/female ratio = 1:2). Fifteen patients (44.1%) had metastatic disease at the time of diagnosis. The most common site of extrahepatic metastasis was lymph nodes (*n* = 6, 17.6%) (Table 1). A majority of the patients (n = 21; 61.8%) had moderately differentiated IHC. ECOG status was 0 in 18 (53%) of the patients, 1 in 11 (32%) and 2 in 1 (3%) of the patients. Four patients’ ECOG status was unknown.

Prior to embolization, 7 (20.5%) patients were treated with chemotherapy, 4 (11.7%) with surgical resection, 7 (20.5%) with a combination of chemotherapy and resection, and 4 (11.7%) patients were treated with chemotherapy and external beam radiation (Table 1). A total of 7 patients (20.5%) underwent LRT (alcohol ablation in 1, radiofrequency ablation in 2 and HAI pump in 4 patients) prior to embolization. Ten patients (29.4%) did not receive any treatment prior to embolization. A variety of systemic and local plus systemic therapy regimens were used (Table 2). The most common was chemotherapy regimen with the combination of gemcitabine and cisplatin (n = 8; 23.5%) based on the ABC-02 study [11]. After embolization, 70.5% of the patients were treated with re-embolization, 28 (82.4%) of the patients continued with at least one chemotherapy regimen and a hepatic artery infusion pump was placed in 5 (14.7%) of the patients. Four patients (11.7%) were treated with external beam radiation and one (2.9%) patient was treated with radioembolization.

The most common indication for embolization was chemorefractory IHC (n = 20; 58.8%), with progression of disease in the liver despite using multiple regimens of chemotherapy. Other indications were the primary treatment option in 10 (29.4%) patients and not tolerating chemotherapy in 3 (8.8%) patients.

Tumor characteristics and patterns are summarized in Table 1. The most common pattern was multifocal (n = 30; 88.2%), and the mean diameter of the largest tumor was 7.6 +/− 3.9 cm. The median number of HAE treatments was 2 sessions (range = 1–5). Two patients (5.8%) were treated with a combination of embolization plus ablation. 

Tumor response is summarized in Table 3. Objective response (OR) was 79.4% (41.1% complete response, CR plus 38.2% partial response, PR) by mRECIST. Two patients (5.8%) had stable disease (SD) and four (11.7%) had progressive disease (PD). Following HAE, 32 (94%) patients experienced local tumor progression. After embolization a total of 24 patients (70.5%) received treatment: chemotherapy in 20 (58.8%), chemotherapy plus radiation therapy in 1 (2.9%), repeat LRT in 3 (8.8%) patients. The type of LRT was alcohol ablation (n = 2, 5.8%) and radioembolization (n = 1, 2.9%). Ten patients (29.4%) did not receive additional treatment after embolization.

**Figure 1:**
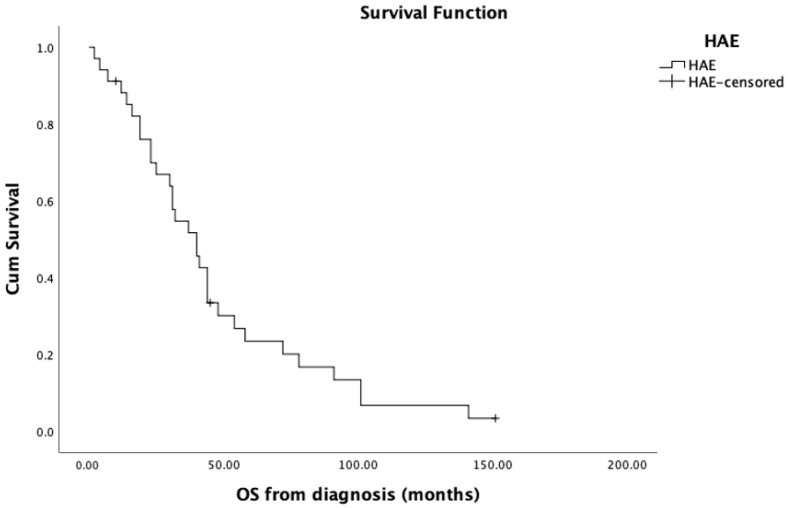
Overall survival in the 34 intrahepatic cholangiocarcinoma patients.

The median OS from initial diagnosis was 40 months (Table 4). The median OS from first embolization was 13 months (SD = 23.9; CI = 13.4–34.1) (Figure 1). Tumor progression was found in the liver in 32 patients (94%) after HAE. Median LT-PFS was 4 months (range: 1–37). OS rates from first embolization at 6 months, 1, and 2 years were 76%, 54% and 38%, respectively. LT-PFS rates from first embolization at 6 months, 1, and 2 years were 42%, 14%, and 10%, respectively.

### 3.2. Factors Associated with Outcome

**Figure 2:**
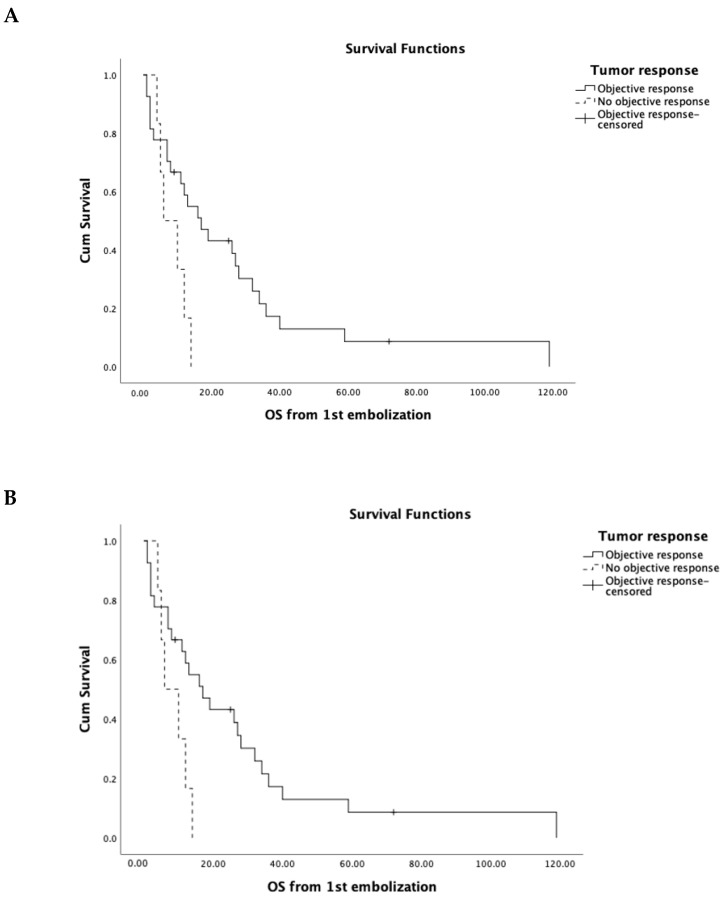
(**A**) Overall survival in patients with objective response compared to patients without objective response. (**B**) Overall survival based on size of the tumors.

1. *Sex*: 68% (n = 23) of the patients were female. The female patients had higher OS compared to male patients (23.67 vs. 16.91 months); however, this difference was not statistically significant (*p* = 0.47).
2. *Pathological grading*: The majority of the patients had moderately differentiated IHC (n = 21). Ten patients had poorly differentiated IHC, and in three patients the grading was unknown. No significant difference in OS was detected between moderately and poorly differentiated ICC (OS in moderately differentiated was 21.8 months vs. 20.3 months in poorly differentiated vs. 20 months in the unknown patients; *p* = 0.9).
3. *Treatment prior to embolization*: A group of 13 patients underwent liver tumor resection prior to embolization, while 21 patients did not undergo any liver tumor resection. Patients that were treated with surgical resection prior to embolization demonstrated significantly higher OS compared to patients that were not treated with resection (34 vs. 14.7 months, respectively, *p* = 0.03).
4. *Presence of extrahepatic metastatic disease at the time of initial diagnosis*: A total of 15 patients presented with metastatic disease at the time of diagnosis (Table 1). These patients demonstrated significantly lower OS post-embolization compared to patients without evidence of metastatic disease (12.9 vs. 31.6; *p* = 0.03).
5. *Indication for embolization*: Patients that were treated as the first line of treatment (n = 10) had higher OS compared to chemorefractory patients (28.5 vs. 17 months). However, this difference was not statistically significant.
6. *Venous invasion*: A total of 7 (20.5%) patients presented with venous involvement at the time of first embolization. The inferior vena cava was involved in 2, the hepatic vein in 1, and the portal vein was invaded in 4 patients. Patients with venous invasion had significantly lower OS compared to patients with no venous involvement (7 vs. 28.6 months; *p* = 0.046).
7. *Tumor response*: Patients who showed OR had significantly longer median OS compared to patients with no OR (OS: 95% CI: 17 months [8.4–25.5] vs. 6 months [1,2,3,4,5,6,7,8,9,10,11,12], *p* = 0.03) (Figure 2A).
8. *Tumor pattern:*
  - *Solitary vs. multifocal:* The majority of patients (88.2%) presented with multifocal disease at the time of embolization. No significant difference was detected between the two groups (29.8 months in solitary tumors vs. 20.7 in multifocal; *p* = 0.45).
  - *Tumor diameter*: Tumors larger than 10 cm (n = 8) were associated with worse OS compared to the other two groups (*p* = 0.0038). Larger than 10 cm tumors had an OS of 7.3 months (Figure 2B).
  - *Percent of liver involvement by the tumor*: Patients with less than 25% (n = 19) tumor involvement in the liver showed significantly longer OS compared to patients with liver involvement of more than 25% (n = 15) (34 vs. 13 months; *p* = 0.0076).
9. *Degree of enhancement on pre-embolization cross-sectional imaging:* Tumors were hypervascular in 12 (35%) patients and hypo- or iso-vascular in 22 (65%). Hypervascular tumors were associated with better OS compared to hypo- and iso-vascular tumors (32.9 months vs. 14.4; *p* = 0.04).

### 3.3. Follow Up and Complications

Mean follow-up period was 32 months. At the time of data analysis, all 34 patients had died. Cause of death was progression of disease. The most common extrahepatic sites for disease progression after embolization were lungs (n = 6, 17.6%) and bones (n = 6, 17.6%).

The patients were hospitalized for an average of 4 days post-embolization (range 2–27). Three patients (8.5%) developed CTCAE Class C complications: post-procedure pancreatitis n = 1 due to non-target embolization and n = 2 requiring prolonged hospitalization course. No Grade 4 or 5 was reported. Classic post-embolization syndrome features were found in 5/34 (14.7%) of patients. Two patients experienced Class B complications (self-limiting ascites and ileus n = 1, slight encephalopathy n = 1) [12].

Statistically significant elevations in Alkaline Phosphatase (ALK), and International Normalized Ratio (INR) were found at the 1-month follow-up labs (ALK: 168 IU/L vs. 187 IU/L, *p* = 0.006; INR: 1 vs. 1.15, *p* =< 0.001). In addition, significant decreases in serum albumin levels were found at the 1-month follow-up labs (4 g/dL vs. 3.7 g/dL, *p* = 0.007).

## 4. Discussion

Unresectable IHC with liver-only or liver-predominate disease is treated with systemic therapy and LRTs. LRTs are either used as first-line or as adjuvant options [7,13,14,15]. However, there is limited evidence on the effectiveness of HAE in treating IHC patients. The median OS in this cohort was 13 months. This is in line with previously published literature examining other types of LRTs [16,17,18,19]. Hyder et al. performed a multicenter study on 198 patients with IHC that were treated with transarterial chemoembolization (cTACE), drug-eluting bead transarterial chemoembolization (DEB-TACE), HAE, and TARE [7]. They reported a median OS of 13.2. They did not detect any statistically significant difference in the OS among different techniques (cTACE, 13.4, DEB-TACE 10.5, HAE 14.3, and TARE 11.3 months). This comparison study was a retrospective study, and unfortunately, to the best of our knowledge, to date there has been no randomized control trial comparing different LRTs for treatment of IHC.

In a systematic review and pooled analysis that focused on LRTs in patients with IHC, 22 cohorts including 1145 patients treated by TACE (and/or HAE) were identified [20]. With data available from 15 studies, the pooled response rate for TACE was 26.3% with strong evidence for heterogeneity among studies. With data available from 7 and 20 studies, the pooled weighted mean PFS was 15.0 months, and the pooled weighted mean OS was 15.9 months, respectively. The response rate was much higher in our study (79.4%). This may be partially explained by the response evaluation criteria that were used, RECIST v1.1 in the review, and mRECIST in the current study. The OS was slightly lower in the current study (13 months). However, patients included in the present study appeared to have more advanced disease, with multifocal disease and metastatic disease rates of 88.2% and 44.1% versus 54.1% and 25.0% in this review, with data available from seven and nine cohorts, respectively [20].

In the current study, patients with hypervascular tumors, measured on pretreatment imaging, demonstrated longer OS compared to non-hypervascular tumors. Multiple studies have demonstrated that HAE is effective in treating hypervascular lesions [9,21,22]. However, similar to colorectal carcinoma, IHC is commonly a “hypovascular” tumor. However, the neovascularization process performed by these tumors makes them susceptible to hypoxia caused by embolization. Tumor vascularity on pretreatment imaging has been previously investigated by multiple investigators [23,24]. Shimohira et al. studied 25 colorectal patients that demonstrated hypervascular lesions on pretreatment imaging [24]. These patients were treated with HAE, and they concluded that HAE appears to be an effective and safe treatment method. Sato et al. reviewed 137 patients with metastatic liver disease from colon, neuroendocrine, and other tumors that were treated with TARE. They divided these tumors into two groups of hypervascular and hypovascular tumors. They did not find any statistical difference in survival between the two groups [23]. This is in contrast to the results of the current study, and it might be due to the mechanism of action of these two different techniques. TARE, particularly the TheraSphere (Boston Scientific Corporation, Marlborough, MA, USA) product, is less embolic and relies on damaging the DNA of the cancer cells. In contrast, HAE results in ischemia; therefore, the damage is caused by inducing hypoxia.

In the current study, patients treated with surgical resection had significantly higher OS compared to patients that were not surgical candidates. Similarly, patients presenting with metastatic disease at the time of diagnosis had worse OS. It is possible that patients that present with metastatic disease have a more aggressive tumor biology compared to those that present as surgical candidates. Further investigation is needed to better answer this question.

In the current study, tumor response and <25% tumor burden presented longer OS. Tumor response has been associated with better OS in other cancers including HCC and breast cancer [25,26]. In a recent study by Ocal et al., the association between OS and OR was explored [26]. Ocal et al. concluded that OR, according to mRECIST, in patients with HCC treated with sorafenib is independent predictor for OS.

The current study has several limitations inherent to its retrospective design. The small sample size (n = 34) does not allow for extensive statistical analysis and might result in skewing of the data. In addition, this study lacks a control group to appropriately compare the efficacy and safety of HAE.

## 5. Conclusions

In conclusion, the findings suggest that HAE is a well-tolerated option for treating IHC, providing 13 months OS. Factors associated with better OS include tumor burden <25%, increased tumor vascularity in target lesion on preprocedure imaging, and surgical resection prior to embolization. Evidence of vascular invasion, metastatic disease at the time of diagnosis, and tumors larger than 10 cm are associated with shorter OS. Larger numbers are needed to verify these data. Given the absence of a control group in this study, some uncertainty regarding the efficacy of HAE as a treatment for IHC exists. Therefore, it is imperative that further studies involving controlled studies be conducted to validate whether HAE indeed constitutes an effective treatment modality.

## Figures and Tables

**Table 1:** Demographic and clinicopathologic characteristics of 34 patients.

Characteristic	Value
Age (mean +/− SD, range)	62 ± 14.03; 31–92
Sex	
Male	11 (32%)
Female	23 (68%)
Metastasis at presentation	
Yes	15 (44.1%)
No	19 (55.9%)
Location of Metastasis	
Lymph node	6 (17.6%)
Lung	1 (3%)
Lung + Lymph node	4 (11.7%)
IVC	1 (3%)
Bone	1 (3%)
Multifocal	2 (5.8%)
Pathology type	
Moderately differentiated	21 (61.8%)
Poorly differentiated	10 (29.4%)
Unknown	3 (8.8%)
Treatment prior to embolization	
Chemotherapy (systemic or arterial infusion)	24 (70.6%)
Hepatic arterial infusion pump	4 (11.7%)
Surgery only	4 (11.7%)
Combination of surgery and chemotherapy	7 (20.5%)
Combination of chemotherapy and external beam radiation	4 (11.7%)
Alcohol ablation and chemotherapy	1 (2.9%)
RFA plus chemotherapy and surgery	2 (5.8%)
None	10 (29.4%)
Tumor characteristics	
Tumor pattern	
Solitary	4 (11.8%)
Multifocal	30 (88.2%)
Largest tumor size (cm)	
Mean, SD	7.6 +/− 3.9
Range	2–17.8
<5 cm	11 (32.3%)
≥5 to ≤10	15 (44.2%)
>10	8 (23.5%)
% of liver involvement	
<25%	19 (55.8%)
25–50%	15 (44.2%)
50–75%	0 (0%)

**Table 2:** Chemotherapy regimen prior to embolization.

Drug/Regimens	Number of Patients (%)
Commonly used systemic chemotherapy	
Gemcitabine and cisplatin	8 (23.5)
Gemcitabine single therapy	6 (17.6)
Other used systemic therapy	
Gemcitabine + capecitabine	3 (8.8)
Gemcitabine + carboplatin	1 (2.9)
Gemcitabine + carboplatin + paclitaxel	1 (2.9)
Gemcitabine + carboplatin + capecitabine	1 (2.9)
Gemcitabine + docetaxel + capecitabine (GTX)	1 (2.9)
Gemcitabine + 5FU	1 (2.9)
Gemcitabine + oxaliplatin	2 (5.8)
Gemcitabine + bevacizumab	1 (2.9)
Gemcitabine + carboplatin + bevacizumab	1 (2.9)
Oxaliplatin + 5FU + Leucovorin	1 (2.9)
Capecitabine + oxaliplatin (XELOX)	1 (2.9)
Capecitabine single therapy	1 (2.9)
Irinotecan single therapy	2 (5.8)
Flurouracil (5FU) single therapy	1 (2.9)
Irinotecan + capecitabine	1 (2.9)
FOLFIRI	2 (5.8)
FOLFOX-6	1 (2.9)
Targeted therapy as a monotherapy after progression on multiple regimens	
Sorafenib	1 (2.9)
Immunotherapy	
Nivolumab	1 (2.9)
Chemotherapy with hepatic arterial infusion pump	
Floxuridine (FUDR) + Mitomycin to side port of the pump	2 (5.8)
Floxuridine (FUDR)	2 (5.8)

**Table 3:** Tumor response, based on mRECIST.

Treatment Response Category	Value	Objective Response (OR)
Complete Response (CR)	14 (41.1%)	27 (79.4%)
Partial Response (PR)	13 (38.2%)	
Stable Disease (SD)	2 (5.8%)	
Progressive Disease (PD)	4 (11.7%)	
Not evaluated (NE)	1 (2.9%)	

**Table 4:** Overall survival and local tumor progression.

OS from 1st embolization	
mean	23.7 ± 23.9 (CI: 13.4–34.1)
median	13 (CI: 7.4–18.5)
range	1–119
OS from diagnosis	
mean, SD	47.4 ± 36.3 (CI: 34.6–60.2)
median	40 (CI: 28.8–51.1)
range	2–151
LT-PFS	
mean, SD	7.7 ± 8.7 (CI: 4.5–10.9)
median	4 (CI: 2.09–5.9)
range	1–37

## Data Availability

The data presented in this study is available on request from the corresponding author.

## References

[1] Shaib Y., El-Serag H.B. (2004). The epidemiology of cholangiocarcinoma. Semin. Liver Dis..

[2] Bridgewater J., Galle P.R., Khan S.A., Llovet J.M., Park J.W., Patel T., Pawlik T.M., Gores G.J. (2014). Guidelines for the diagnosis and management of intrahepatic cholangiocarcinoma. J. Hepatol..

[3] Nathan H., Pawlik T.M., Wolfgang C.L., Choti M.A., Cameron J.L., Schulick R.D. (2007). Trends in survival after surgery for cholangiocarcinoma: A 30-year population-based SEER database analysis. J. Gastrointest. Surg..

[4] Rizvi S., Khan S.A., Hallemeier C.L., Kelley R.K., Gores G.J. (2018). Cholangiocarcinoma—Evolving concepts and therapeutic strategies. Nat. Rev. Clin. Oncol..

[5] Cunningham S.C., Choti M.A., Bellavance E.C., Pawlik T.M. (2007). Palliation of hepatic tumors. Surg. Oncol..

[6] Burger I., Hong K., Schulick R., Georgiades C., Thuluvath P., Choti M., Kamel I., Geschwind J.F.H. (2005). Transcatheter arterial chemoembolization in unresectable cholangiocarcinoma: Initial experience in a single institution. J. Vasc. Interv. Radiol. JVIR.

[7] Hyder O., Marsh J.W., Salem R., Petre E.N., Kalva S., Liapi E., Cosgrove D., Neal D., Kamel I., Zhu A.X. (2013). Intra-arterial therapy for advanced intrahepatic cholangiocarcinoma: A multi-institutional analysis. Ann. Surg. Oncol..

[8] Jordan O., Denys A., De Baere T., Boulens N., Doelker E. (2010). Comparative study of chemoembolization loadable beads: In vitro drug release and physical properties of DC bead and hepasphere loaded with doxorubicin and irinotecan. J. Vasc. Interv. Radiol. JVIR.

[9] Maluccio M., Covey A.M., Gandhi R., Gonen M., Getrajdman G.I., Brody L.A., Fong Y., Jarnagin W., D’Angelica M., Blumgart L. (2005). Comparison of survival rates after bland arterial embolization and ablation versus surgical resection for treating solitary hepatocellular carcinoma up to 7 cm. J. Vasc. Interv. Radiol. JVIR.

[10] Llovet J.M., Di Bisceglie A.M., Bruix J., Kramer B.S., Lencioni R., Zhu A.X., Sherman M., Schwartz M., Lotze M., Talwalkar J. (2008). Design and endpoints of clinical trials in hepatocellular carcinoma. J. Natl. Cancer Inst..

[11] Valle J., Wasan H., Palmer D.H., Cunningham D., Anthoney A., Maraveyas A., Madhusudan S., Iveson T., Hughes S., Pereira S.P. (2010). Cisplatin plus gemcitabine versus gemcitabine for biliary tract cancer. N. Engl. J. Med..

[12] Sacks D., McClenny T.E., Cardella J.F., Lewis C.A. (2003). Society of Interventional Radiology clinical practice guidelines. J. Vasc. Interv. Radiol. JVIR.

[13] Boehm L.M., Jayakrishnan T.T., Miura J.T., Zacharias A.J., Johnston F.M., Turaga K.K., Gamblin T.C. (2015). Comparative effectiveness of hepatic artery based therapies for unresectable intrahepatic cholangiocarcinoma. J. Surg. Oncol..

[14] Bragazzi M.C., Venere R., Ribichini E., Covotta F., Cardinale V., Alvaro D. (2023). Intrahepatic cholangiocarcinoma: Evolving strategies in management and treatment. Dig. Liver Dis..

[15] Brown K.T., Do R.K., Gonen M., Covey A.M., Getrajdman G.I., Sofocleous C.T., Jarnagin W.R., D’Angelica M.I., Allen P.J., Erinjeri J.P. (2016). Randomized Trial of Hepatic Artery Embolization for Hepatocellular Carcinoma Using Doxorubicin-Eluting Microspheres Compared With Embolization With Microspheres Alone. J. Clin. Oncol..

[16] Gusani N.J., Balaa F.K., Steel J.L., Geller D.A., Marsh J.W., Zajko A.B., Carr B.I., Gamblin T.C. (2008). Treatment of unresectable cholangiocarcinoma with gemcitabine-based transcatheter arterial chemoembolization (TACE): A single-institution experience. J. Gastrointest. Surg..

[17] Kuhlmann J.B., Euringer W., Spangenberg H.C., Breidert M., Blum H.E., Harder J., Fischer R. (2012). Treatment of unresectable cholangiocarcinoma: Conventional transarterial chemoembolization compared with drug eluting bead-transarterial chemoembolization and systemic chemotherapy. Eur. J. Gastroenterol. Hepatol..

[18] Park S.Y., Kim J.H., Yoon H.J., Lee I.S., Yoon H.K., Kim K.P. (2011). Transarterial chemoembolization versus supportive therapy in the palliative treatment of unresectable intrahepatic cholangiocarcinoma. Clin. Radiol..

[19] Rafi S., Piduru S.M., El-Rayes B., Kauh J.S., Kooby D.A., Sarmiento J.M., Kim H.S. (2013). Yttrium-90 radioembolization for unresectable standard-chemorefractory intrahepatic cholangiocarcinoma: Survival, efficacy, and safety study. Cardiovasc. Interv. Radiol..

[20] Edeline J., Lamarca A., McNamara M.G., Jacobs T., Hubner R.A., Palmer D., Groot Koerkamp B., Johnson P., Guiu B., Valle J.W. (2021). Locoregional therapies in patients with intrahepatic cholangiocarcinoma: A systematic review and pooled analysis. Cancer Treat. Rev..

[21] Fiore F., Del Prete M., Franco R., Marotta V., Ramundo V., Marciello F., Di Sarno A., Carratù A.C., de Luca di Roseto C., Colao A. (2014). Transarterial embolization (TAE) is equally effective and slightly safer than transarterial chemoembolization (TACE) to manage liver metastases in neuroendocrine tumors. Endocrine.

[22] Strosberg J.R., Choi J., Cantor A.B., Kvols L.K. (2006). Selective hepatic artery embolization for treatment of patients with metastatic carcinoid and pancreatic endocrine tumors. Cancer Control.

[23] Sato K.T., Omary R.A., Takehana C., Ibrahim S., Lewandowski R.J., Ryu R.K., Salem R. (2009). The role of tumor vascularity in predicting survival after yttrium-90 radioembolization for liver metastases. J. Vasc. Interv. Radiol. JVIR.

[24] Shimohira M., Sato Y., Yasumoto T., Kodama Y., Masada T., Inaba Y., Yamakado K. (2021). Arterial Embolization Using Microspheres for Hypervascular Liver Metastases Refractory to Standard Treatments: A Multicenter Prospective Clinical Trial. Cardiovasc. Interv. Radiol..

[25] Ridouani F., Soliman M.M., England R.W., Hsu M., Moskowitz C.S., Doustaly R., Sofocleous C.T., Boas F.E., Yarmohammadi H., Deipolyi A.R. (2021). Relationship of radiation dose to efficacy of radioembolization of liver metastasis from breast cancer. Eur. J. Radiol..

[26] Öcal O., Schinner R., Schütte K., de Toni E.N., Loewe C., van Delden O., Vandecaveye V., Gebauer B., Zech C.J., Sengel C. (2022). Early tumor shrinkage and response assessment according to mRECIST predict overall survival in hepatocellular carcinoma patients under sorafenib. Cancer Imaging.

